# Incidental pheochromocytoma presenting with sublaboratory findings in asymptomatic surrenal masses: a case report

**DOI:** 10.1186/1757-1626-1-10

**Published:** 2008-05-25

**Authors:** Mesut Ozkaya, Mehmet Fatih Yuzbasioglu, Ertan Bulbuloglu, Sevgi Bakaris, Hafize Oksuz, Kadir Gisi, Ahmet Onder

**Affiliations:** 1 Departments of Endocrinology and Metabolism, Sutcu Imam University Medical Faculty, Kahramanmaras, Turkey; 2 Departments of General Surgery, Sutcu Imam University Medical Faculty, Kahramanmaras, Turkey; 3 Departments of Pathology, Sutcu Imam University Medical Faculty, Kahramanmaras, Turkey; 4 Departments of Anesthesia and reanimation, Sutcu Imam University Medical Faculty, Kahramanmaras, Turkey

## Abstract

**Introduction:**

Adrenal incidentaloma can be described as adrenal lesions that are incidentally diagnosed during abdominal laparotomy or any abdominal screening without prior suspicion of adrenal disease. It is important to diagnose adrenal lesions to learn if they are hormonally active or malignant. The most common clinical sign of pheochromocytoma is sustained or paroxysmal hypertension, and the most common symptoms are headache, excessive truncal sweating, and palpitation. In some cases, the clinical symptoms are not clear. Roughly 70% of adrenal incidentalomas are non-functional. A small group of 5–7% of the functional ones (30%) may exist as pheochromocytoma. Ten percent of pheochromocytoma cases are diagnosed incidentally during computed tomography (CT) or magnetic resonance imaging (MRI) screenings for other reasons. The most frequent symptom of the pheochromocytoma is hypertension, and 90–100% of cases exhibit it. The literature indicates that incidental pheochromocytoma cases that are smaller than 1 cm have no clinical symptoms. Rarely, some large pheochromocytoma cases do not show any clinical symptoms, and it is difficult to diagnose very small ones.

**Case presentation:**

A 45-year-old male patient experienced an epigastric ache and distended stomach for 7 years. The serum cortisol level was 19.2 ng/dL (normal range: 5–20 ng/dL), and urinary free cortisol excretion was 25.00 μg/24 h (normal range:10.00–100.00 μg/24 h). The serum basal level of adrenocorticotropic hormone (ACTH) was 21 pg/mL (normal range: 9 to 52 pg/mL). Plasma cortisol was under 1.00 μg/dL after low dose (1 mg) overnight dexamethasone suppression test. 24 hours urinary catecholamines level were vanil mandilic acid (VMA) 8.90 mg/day (normal range, 3 to 90 mg/day), metanefrin 330 μg/day (normal range, 52 to 341 μg/day), epinefrin 13 μg/day (normal range, 2 to 24 μg/day), norepinefrin 41 μg/day (normal range; 15 to 100 μg/day). During abdominal ultrasonography (USG), a tumor was diagnosed in the right perirenal space. A regular-shaped mass (dimension 36 × 35 × 35 mm) with a homogeneous and solid structure was diagnosed in CT. The density of the mass was 80 Hounsfield units (HU) in postcontrast CT. The patient was given a diagnosis of a non-functional adrenal incidental lesion, underwent a right adrenalectomy. Histopathological data correlated with pheochromocytoma as well

**Conclusion:**

Pheochromocytoma can be diagnosed by establishing an increase in catecholamines and metabolites in the plasma and urine. The level of catecholamines and metabolites in the plasma and urine provide 95% of the evidence of the disease. Because the dimensions of the lesion were large and the HU was very clear, the patient was underwent surrenalectomy. During laboratory investigation, there was no evidence of abnormality; we, therefore, think that these cases can be named sublaboratory pheochromocytoma.

## Introduction

Adrenal incidentaloma can be described as adrenal lesions that are incidentally diagnosed during abdominal laparotomy or any abdominal screening without prior suspicion of adrenal disease. This definition does not include adrenal lesions that are diagnosed for oncological metastasis. In the last 30 years, case diagnosis of adrenal incidentaloma increased about 20 times because of technological development. It is important to diagnose adrenal lesions if the lesions are hormonally active or malignant. The number of adrenal incidental cases increase with age and application technique. Statistical autopsy data suggests that these lesions occur in 1% of people at age 30, but 7% at age 70 with no difference in gender [1].

Pheochromocytoma is a tumoral disease that originates from chromaffin cells in the sympathoadrenal system. These tumors secrete epinephrine, norepinephrine, and in some cases, dopamine and catecholamines. They secrete other active peptides and cause symptoms of hypertension. Because pheochromocytoma is a rare disease, it is present in only 0.1–0.2% of hypertension patients. Although there is no difference in age or gender, occurrence increases in the 4th and 5th decades. Most pheochromocytoma cases localize to the abdomen (90%), and 90% of abdomen-localized cases are in the adrenal glandulas. The most common clinical sign of pheochromocytoma is a sustained or paroxysmal hypertension, and the most common symptoms are headache, excessive truncal sweating, and palpitation. Angina pectoris, abdominal pain, queasiness, anxiety, and paleness are other symptoms of it as well. In some cases, clinical symptoms are not clear until they are diagnosed incidentally during computed tomography (CT) or magnetic resonance imaging (MRI) [2,3].

Roughly 70% of adrenal incidentalomas are non-functional. A small group of 5–7% of the functional ones (30%) may exist as pheochromocytomas [4]. We review the literature because our case had no preoperative clinical or laboratory findings and was diagnosed postoperatively with a pathologic investigation.

## Case

### Case presentation

A 45-year-old male patient experienced an epigastric ache and distended stomach for 7 years. During abdominal ultrasonography (USG), a tumor was diagnosed in the right suprarenal fossa. He did not use any drugs and there was no characteristic of past medical and family illness history. He was 1.74 cm and weighted 81 kg, with a body mass index (BMI) of 26.5 kg/m^2^. His right hand tension arteriel was 110/70 mmHg and his left hand tension arterial was 115/70 mmHg; when standing, these values were 110/70 and 110/70 mmHg, respectively. Heart rate was 80 beats/minute, and no pathologic data was found in the system examination. The laboratory data showed that hemogram and urine findings were normal. His lipid profile was normal. Other findings were as in Table 1.

**Table 1:** Laboratory findings.

	**Levels**	**Normal Range**
Glucose	87 mg/Dl	70 to 100 mg/dL
BUN	20 mg/dL	7 to 18 mg/dL
Creatinine	0.9 mg/dL	0 to 1.3 mg/dL
Na	144 mmol/L	136 to 145 mmol/L
K^+^	4 mmol/L	3.5 to 5.5 mmol/L
Ca+^2^	9.6 mg/dL	8.5 to 10.1 mg/dL
Cl	96 mmol/L	96 to 110 mmol/L
P	4.7 mg/dL	2.5 to 4.9 mg/dL
AST	13 U/L IU/L	15 to 37 IU/L
ALT	33 U/L	30 to 65 IU/L

Plasma cortisol was under 1.00 μg/dL after low dose (1 mg) overnight dexamethasone suppression test. 24 hours urinary catecholamines level were vanil mandilic acid (VMA) 8.90 mg/day (normal range, 3 to 90 mg/day), metanefrin 330 μg/day (normal range, 52 to 341 μg/day), epinefrin 13 μg/day (normal range, 2 to 24 μg/day), norepinefrin 41 μg/day (normal range; 15 to 100 μg/day). Hormonal levels were as in Table 2.

**Table 2:** Hormonal levels

**Hormonal Tests**	**Levels**	**Normal Range**
TSH	1.53 μIU/mL	0.4 to 4.0 μIU/mL
LH	8.97 IU/L	3.6 to 17.1 IU/L
PRL	8.74 ng/mL	1.6 to 18.8 ng/mL
Insulin	5.75 μU/ml	3 to 20 μU/ml
PTH	8.74 pg/ml	7 to 11 pg/ml
Calcitonin	3.2 ng/L	< 8 ng/L
Total testosterone	332 ng/dl	265 to 800 ng/dl
Plasma aldosterone	11 ng/dl	5 to 20 ng/dl
DHEA-S	214 μg/dl	59 to 492 μg/dL
Cortisol	19.2 ng/dL	5–20 ng/dL
Urinary free cortisol excretion	25.00 μg/24 h	10.00–100.00 μg/24 h
Serum basal level of ACTH	21 pg/mL	9 to 52 pg/mL

### Abdominal USG

There was a 34 × 26 mm solid lesion in the right perirenal space.

### Abdominal CT

**Figure 1:**
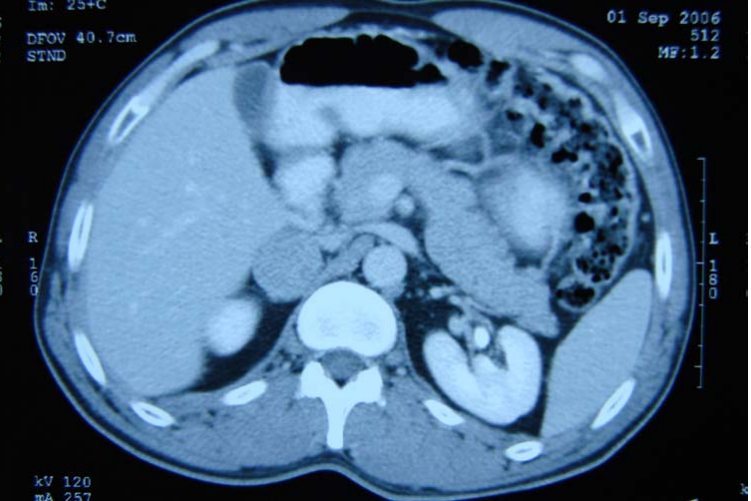
Mass in Abdominal CT.

Liver parenchyma was homogeneous. In the right lobe of the liver, there was a two millimeter subcapsular calcification. A regular-shaped mass (dimension 36 × 35 × 35 mm) with a homogeneous and solid structure was diagnosed at the right suprarenal fossa. The density of the mass was 80 Hounsfield units (HU) in postcontrast CT and 40 HU without contrast CT [Fig. 1].

**Figure 2:**
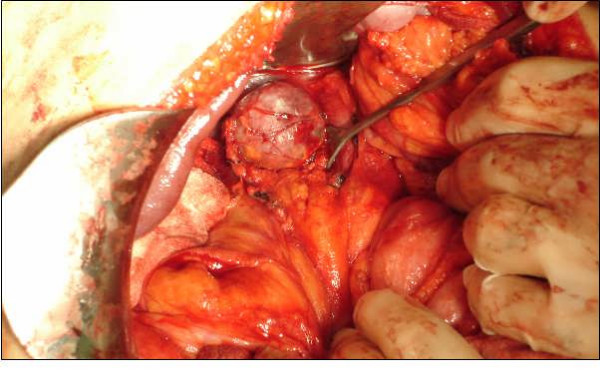
Mass in intraoperative view.

**Figure 3:**
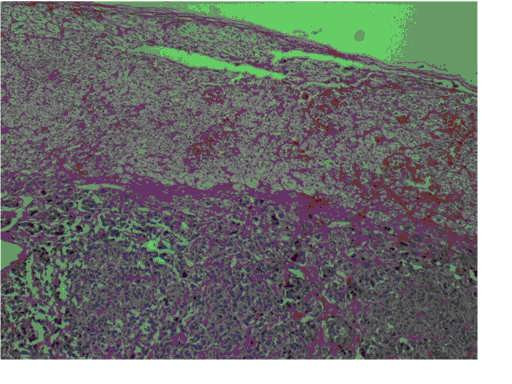
Pathologic view. Stain: (H&E). Magnification: ×40.

After all these examinations, the patient was given a diagnosis of a non-functional adrenal incidental lesion. Because it was 80 HU in contrast CT, 40 HU without contrast CT, and 36 × 35 × 35 mm, the patient underwent a right adrenalectomy [Fig. 2]. There were no clinical problems during, before, or after the surgery. The pathology examination confirmed the mass as being pheochromocytoma [Fig 3]. The patient was discharged after 5 days.

## Discussion

The number of incidentally found adrenal lesions is growing. The most important problem in appraising adrenal incidental cases is diagnosing the lesion as benign or malignant. To distinguish malign and benign lesions, screening methods must be used. CT is an ideal screening method for the adrenal glandula and the most helpful technique for distinguishing cysts, bleedings, and myelolipomas. On the other hand, MRI is successful in distinguishing adrenal adenomas, carcinomas, and metastatic masses. But CT has gained widespread acceptance as the technique of choice for distinguishing between adrenal adenomas and metastasis [5].

Adenomas frequently contain sufficient intracytoplasmic fat to produce lower attenuation values. With an increasing proportion of lipid mass, the density will be lower. Although the lipid proportion is high in benign cases, it is lower in malignant ones. No adrenal malignancy with a density of 0 HU has been reported in the literature. It is suggested that 4–20 HU density cases must be suspicious and patients in these cases undergo MRI. The cases in which the density is higher than 20 HU are rated as malign in some other studies [6]. In some benign cases (especially acute phase bleeded), tomogrophic density is very high. Pheochromocytomas are often well-defined masses with attenuation values similar to those of muscle tissue, measuring approximately 30–40 HU. The density of the mass in our case was 80 HU in postcontrast CT and 40 HU without contrast CT.

The dimensions of lesion and a history of cancer are important factors for distinguishing whether masses are benign or not. While the carcinoma prevalence is 2 % for the masses smaller than 4 cm, it is 6 % for the ones 4–6 cm and 25 % for those bigger than 6 cm. It is impossible to morphologically distinguish adrenal metastases from benign masses. The most reliable technique for distinguishing these two masses involves conducting a percutan biopsy after elimination of the diagnosis of pheochromocytoma. If the mass is pheochromocytoma, percutan biopsy will cause a hypertension crisis. Biopsy is not an adequate technique for distinguishing adrenocortical adenomas and adrenocortical carcinomas [7]. While our patient's CT investigation density was high, he underwent surgery since the biopsy is invasive. We did not perform a biopsy for the case.

The second important question in adrenal incidentaloma is whether a lesion is functional or not. No functional scanning is needed for myelolipoma and adrenal cysts, which were diagnosed with screening methods alone. According to the increase in the sensitivity of the endocronologic tests used for differentiation diagnosis, the prevalence of the subclinical syndrome and/or a silent preclinical one is increased in these cases.

About 5–10 % of the adrenal incidental cases are subclinical instances of Cushing syndrome. In all cases, Cushing screening should be conducted with a 1 mgr Dexametazone test. In this test, the limit for the pressured plasma cortizol level is 1.8 μg/dL [8]. In our cases, there was suppression after 1 mgr Dexametasone suppression. Free cortizol levels in urine were normal after 24 hours, and so we concluded that there was no pathology in glucocorticoid function.

The level of the plasma potassium and renin-aldosterone must be checked in hypertensioned adrenal incidental cases. If the level of renin-aldosterone activity is greater than 30 and the level of plasma aldosterone is higher than 20 ng/dl, there is an autonomic aldosterone secretion. Roughly 80% of the primary aldosteronism are adenomas and 1% of the cases are adrenocortical carcinomas. In cases of adrenocortical adenoma with primer aldosteronism, the dimensions of the adenomas are typically small [9]. Because there was no arterial hypertension in our case and the level of aldosterone was normal, we did not attribute diagnosis to an increase of mineralocorticoid function.

Tumors producing androgens are rare. They may be malignant or benign, and clinically, they have virilization effects. DHEA-S provides the most important evidence for the secretion of adrenal androgen. The level of DHEA-S should be measured in adrenal incidentaloma cases. There was no increase in androgen function due to the lack of hyperandrogenic findings, and the levels of DHEA-S were normal in our case.

Pheochromocytoma is a rare catecholamine secreting tumor derived from chromaffin cells. It is thought that 5–7 % of functional adrenal incidental masses may be pheochromocytomas. Although pheochromocytoma is not one of the main causes of hypertension, it will be fatal if not controlled. About 10 % of pheochromocytoma cases are diagnosed incidentally during CT or MRI that are screened for other reasons [10]. Fifty percent of cases are diagnosed by autopsy, and this shows that the disease is seen frequently as well. Sometimes, prediagnosed pheochromocytoma is proposed, but rarely can the diagnosis really be pheochromocytoma. In one series of 300 cases, only one patient had pheochromocytoma [11]. On the other hand, the proportion of malignant pheochromocytoma cases is 2.5–14 %, and these findings are diagnosed with metastases or local invasion [3].

The literature states that incidental pheochromocytoma cases that are smaller than 1 cm have no clinic symptoms [12]. Rarely, some large pheochromocytoma cases do not show any clinical symptoms [13]. It is difficult to diagnose very small ones. Pheochromocytoma can be diagnosed during investigation of other diseases or adrenal incidentaloma. One line of research from the Mayo Clinic shows that 15 of the 150 pheochromocytoma cases are diagnosed during abdominal CT taken for other reasons [10]. One of the important signs of silent pheochromocytomas is the occurrence of hypertension attacks during surgery or anesthesia [14]. Our case showed no hypertension or hypertension attacks in his history. Physical examination revealed no hypertension-related symptoms. Furthermore, he did not have any complaints except epigastric bloating. Additionally, there were no abnormalities in ecocardiography or the ECG. Consequently, pheochromocytoma was not considered.

Pheochromocytoma can be diagnosed by establishing the increase of catecholamines and metabolites in the plasma and urine. The level of catecholamines and metabolites in the plasma and urine provides 95 % of the evidence for the disease. Free catecholamines (epinephrine or norepinephrine) or metabolites (metanephrine, normetanephrine, or VMA) can be measured in the urine, and the levels of metanephrine or catecholamines can be measured from the plasma. Measurement of metanephrine in the urine is the most useful test [14]. If the level of catecholamines in the urine 2 or 3 times as high as normal, a pheochromocytoma may be present. There was no excess of catecholamines in the plasma or urine in our case.

The dimension of the mass was > 3 cm but the HU was up to 40 HU. Our patient underwent surrenalectomy even though there was no incidental pheochromocytoma. Laboratory findings support the absence of a pheochromocytoma. Since the operation material of the patient showed some findings related to pheochromocytoma, the patient was scanned for multiple endocronological neoplasias (MEN) in the postoperative period. Calcitonin and PTH levels detected for screening MEN. No clinical or diagnostical findings have been observed.

All incidental adrenal cases must be examined very carefully, because the chance of mortality is high when they are not diagnosed. Incidental pheochromocytoma cases have nonhomogenic, cystic, and hemoragic images in screening methods. Because our case was homogeneous, we did not consider pheochromocytoma as a diagnosis for our case. The image of a bilateral surrenal incidental lesion is one of the most important symptoms of pheochromocytoma. The image of the left adrenal was normal in our case.

## Conclusion

During the laboratory investigation, there was no evidence of abnormalities. We, therefore, conclude that these cases can be regarded as sublaboratory pheochromocytomas. However, further studies of more cases must be done to clarify this subject.

## Abbreviations

CT: Computed tomography; MRI: Magnetic resonance imaging; BMI: Body mass index; USG: Ultrasonography; VMA: Vanil mandilic acid (VMA); PTH: Parathyroid hormone; ACTH: Adrenocorticotropic hormone; HU: Hounsfield Unit; DHEA-S: Dehidroepiandrosteron sulfate; ECG: Electrocardiography.

## Competing interests

The authors declare that they have no competing interests.

## Authors' contributions

MO was involved the clinical preparation and prediagnosis of the patient, SB was involved the preparation of pathological findings. All other authors conducted the operation and prepared the manuscript.

## Patient Consent

We have obtained written, informed patient consent for publication of the report and any accompanying images.
